# Modifying Polylactide with Powdered Cork Filler

**DOI:** 10.3390/ma18245606

**Published:** 2025-12-13

**Authors:** Mariusz Fabijański, Jacek Garbarski, Zbigniew Szymaniak

**Affiliations:** Polymer Processing Department, Faculty of Mechanical and Industrial Engineering, Warsaw University of Technology, 85 Narbutta Street, 02-524 Warsaw, Poland; jacek.garbarski@pw.edu.pl (J.G.); zbigniew.szymaniak@pw.edu.pl (Z.S.)

**Keywords:** polylactide, compatibilizer, cork filler, mechanical and physical properties

## Abstract

The paper presents the results of experimental testing of a PLA-based composite, modified with powdered cork and a compatibilizer. The purpose of applying these additives was to evaluate their influence upon the physical, structural and functional properties of the obtained material. Specimens with various cork and compatibilizer contents were analyzed to evaluate the synergic interaction between the polymer base and the filler. The tests of the mechanical properties, water absorption and FTIR analysis were carried out. The results confirmed that the cork filler improved the PLA-based composite both in terms of the utility and ecological aspects. Despite a certain mechanical deterioration, the properties remain fully acceptable for packaging applications. Also, the improvement of hardness at higher cork content was observed, which points to effective phase interaction and a good adherence of the components. The FTIR spectroscopy confirmed chemical stability of the base and the lack of unwanted degradation reactions. The obtained composite is an innovative, biodegradable polymer material that utilizes natural waste in a way which is both economic and environmentally friendly. The obtained results point to a high application potential of this kind of composite, mainly in the packaging industry and in the field of ecological utility materials.

## 1. Introduction

In recent years the demand for biodegradable materials has rapidly risen. This is due to the utilization problems concerning polymer packaging, which are usually disposable and short-span products. Their contribution to the total mass of polymer waste is high [1,2,3,4,5]. Generally, the utilization of polymer waste brings about serious problems mainly due to their variety and contamination. It concerns packaging where the ratio of surface (thus the degree of contamination) to mass is very high. From the pure economic point of view the use of polymer waste regranulate is unprofitable. The original material bought directly from the organic synthesis manufacturer is cheaper. That is the reason why biodegradable polymers are of such an interest, because recycling in the traditional meaning is not required. Those materials decompose either in the natural environment or in industrial composting plants [6,7,8,9,10,11].

As with any other material, they have, however, certain disadvantages. One of them is their high price compared to similar traditional polymers (polypropylene, polyethylene) [8,12,13,14,15]. One of the possible solutions is the application of mineral or organic fillers. Not always do they improve mechanical properties; on the contrary, they often worsen them. The process should be carried out in such a way as to obtain the minimum required mechanical properties at the maximum possible filling [16,17,18,19,20,21,22,23].

Powdered cork, which is this kind of filler, is manufactured from industrial waste [24,25]. The cork filler stabilizes the dimensions of the item, reduces thermal conductivity and extends the lifespan of the final product a bit so that it does not decompose too fast. It is characterized by low density, five times lower than that of water, softness, elasticity and resistance to biological and chemical agents. Thanks to its structure it dampens vibrations, its electrical conductivity is zero and it is impermeable to liquids and gases. Cork products such as liners have antistatic properties. Cork does not change its properties at various temperatures and humidity [26,27,28,29,30,31,32,33,34].

The investigation on cork-filled polymers showed a dramatic drop in mechanical properties even at small quantities of the filler [35,36,37,38,39]. This is why, in this work, an attempt was undertaken to prepare a polymer composite based on biodegradable polylactide [40,41,42,43,44]. PLA is a thermoplastic polymer, produced from renewable raw materials, and is totally biodegradable. Its properties are very much like the ones of polystyrene. It is commonly used for 3D printing because of its ease of printing and low melting temperature. Currently much research to modify this material is being conducted, including application of powdered cork [45,46,47,48,49].

The main purpose of this paper was the evaluation of properties of new polymer composites based on PLA in which various contents of powdered cork were applied. The innovation consists of introducing into the material a special modifier (compatibilizer), which is a mixture of various biodegradable materials, mainly thermoplastic starch [50,51,52,53,54]. The novelty challenge is the elimination of dramatic drop of strength, which usually accompanies high organic filler content and essential improvement of adhesion between hydrophobic polymer base and the hydrophilic cork particles. The modifier ensures even distribution of the filler during injection or extrusion which is a key factor in obtaining repeatable utility properties [55,56,57,58,59].

## 2. Materials and Methods

Polylactide manufactured by Nature Works (Plymouth, MN, USA) of the commercial name IngeoPolymer 3260HP was used in this work [60]. This material is intended for injection and extrusion. Its Melt Flow Rate (MFR) is 65 g / 10 min (210 °C, 2.16 kg). It is a highly efficient, polycrystalline polylactide acid, able to crystalize during processing. That means, in turn, high deflection temperature under load (Marten’s test). In crystalline form it is semitransparent and is suitable for products of longer exploitation time. In this form, however, it is not biodegradable. That is why, to obtain the fully amorphous (biodegradable) phase, the injection molds should work at temperatures 80–130 °C [60].

The research work used natural cork granulate supplied by CORKPOL (CAS No. 61789.98-8; CORKPOL, Ożarów Mazowiecki, Poland). The basic raw material for its production is cork oak, obtained through the process of periodic peeling of the bark. Its grain fraction covers the dimensional range 0.2–0.8 mm, which ensures good processing properties and easy distribution in the polymer base. Granule density ranges from 45 to 200 kg/m^3^, influenced by environmental and biological conditions, which naturally lead to varying porosity and cell density within the cortex. This directly translates into the final, highly variable density of the material. Despite this variability, cork is an exceptionally lightweight material characterized by a highly porous structure. Its characteristic brown color and weak, natural smell also belong to its physical properties [61].

The most important feature of the material is its high thermal stability, confirmed by its ignition temperature above 300 °C, which enables its processing at the standard processing ranges of temperature for most polymers. Despite the hydrophilic character of the cork resulting from the presence of a mesh structure, drying at the temperature 60–70 °C for 2–4 h prior to processing is recommended. This eliminates the possible surface humidity [61].

To improve the adhesion between the hydrophobic polymer base (PLA) and the hydrophilic surface of the cork filler, a special modifier manufactured by Grupa Azoty (Poland, Tarnów) was applied. It is a powder of grain size like that of the cork filler. It has the form of a low viscous mixture of biodegradable polymers consisting mainly of thermoplastic starch [62]. The modifier called in the jargon as “glue” plays a key role as a interfacial compatibilizer. Its function consists of migration in the surface of the cork particles, where it creates a flexible intermediate layer that reduces interfacial tension, thus increasing adhesion at the interface base-filler. Also, due to its liquefying properties, the modifier homogenizes and makes the distribution of the filler in the polymer melt easier, which finally improves the homogeneity of the composite structure and its mechanical strength [62].

The specimens were injection molded with the industrial injection machine UT90 (Ponar Żywiec, Żywiec, Poland). The machine was equipped with a 5-point, double lever clamping system; direct drive of the screw with the help of high torque hydraulic motor ensured accurate control of processing parameters. The entire testing stand was completed by integrating the machine with peripheral devices such as a special injection mold with exchangeable forming units, enabling manufacturing of both paddles and beams, thermostat, laboratory dryer KC100/200, high accuracy electronic scales DAR wag and a grinder for milling polymer waste.

Based on the recommendation of PLA, cork and “glue” manufacturers, the components were dried at 60 °C for 8 h [60,61,62]. Humidity had a disadvantageous influence on the quality of the specimens. Then the mixtures were prepared. The contents of the polymer composition are presented in Table 1.

To check the sole interaction between the PLA and the “glue”, two compositions of different modifier content recommended by the manufacturer (3% wt. and 5% wt., without cork) were prepared. Finally, the 3% wt. content was selected, as were three compositions with cork prepared (Table 1).

The compositions were mixed in the rotation mixer. Then the primary tests were run on the injection machine. Some readjustment of the temperature of the barrel zones was necessary. Plasticizing polymer with the filler generated heat. Thus, the temperature in all zones had to be lowered. The final injection parameters are given in Table 2.

All produced specimens (paddles and beams) underwent 48 h conditioning at 23 °C and relative humidity 50% prior to testing. The tensile stretching test was carried out according to the standards ISO 527-1 and ISO 527-2. For mechanical measurements, strength tester Fu1000e (Heckert, Germany) of the maximum tensile force 10 kN was used. Force and elongation were measured at the clamp speed 2 mm/min.

Impact strength was measured using the pendular hammer (Charpy ISO 179-1) manufactured by Wolfgang Ost (Solingen, Germany) of maximum impact energy 4 J, using unnotched specimens. Hardness was measured with the Shore method, by means of electronic hardness meter XINGWEIQIANG (Shenzen, China), equipped with the scale 0–100 D, in accordance with ISO 868.

The water absorption measurement was carried out, according to ISO 62. During the test, which lasted one month, 10 paddle-shaped specimens were tested. The procedure started with weighing the specimens and placing them in a container filled with water. In regular 24 h intervals the specimens were taken out and a two-stage process of removing water from their surface was begun. First the specimens were placed vertically upright to drain the surplus of water, which took 10 min. Then they were moved to a dry fabric, where they were left for 30 min to make the remaining water evaporate. Finally, the specimens were weighed with a fine scale of the accuracy 0.001 g and the measuring range 1–200 g and again submerged in water, repeating the measurements for the entire test period.

Fourier transform infrared (FTIR) spectra were measured using a Thermo Fisher iS5 spectrometer (Thermo Scientific, Madison, WI, USA), following the manufacturer’s recommended procedure in the device’s technical documentation. To obtain representative data for the analyzed PLA composite samples with natural cork, the Attenuated Total Reflectance technique was used, enabling direct surface measurement without the need for prior sample preparation. Measurement parameters were selected to enable efficient analysis of both the polymer matrix (PLA) and the organic filler (cork). Spectral resolution was set at 4 cm^−1^, which is optimal for clearly separating key absorption bands—in particular, the band corresponding to the C=O bond characteristic of PLA and the bands associated with the presence of cellulose and lignin in the cork structure. The aim of the measurements was to identify the chemical composition of the composites and to assess potential interfacial interactions between the polymer matrix and the organic filler.

## 3. Results and Discussion

**Figure 1:**
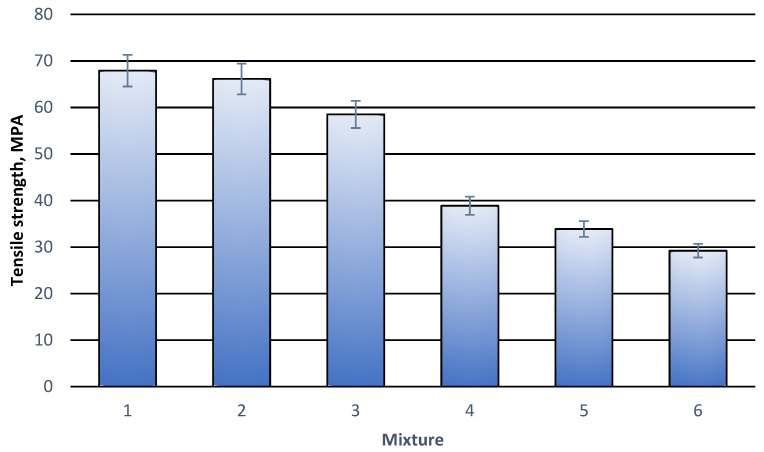
Tensile stress at break for individual polymer compositions. (**1**) 100% wt. PLA; (**2**) 97% wt. PLA + 3% wt. modifier; (**3**) 95% wt. PLA + 5% wt. modifier; (**4**) 92% wt. PLA + 3% modifier + 5% wt. cork filler; (**5**) 87% wt. PLA + 3% wt. modifier + 10% wt. cork filler; (**6**) 82% wt. PLA + 3% modifier + 15% wt. cork filler.

**Figure 2:**
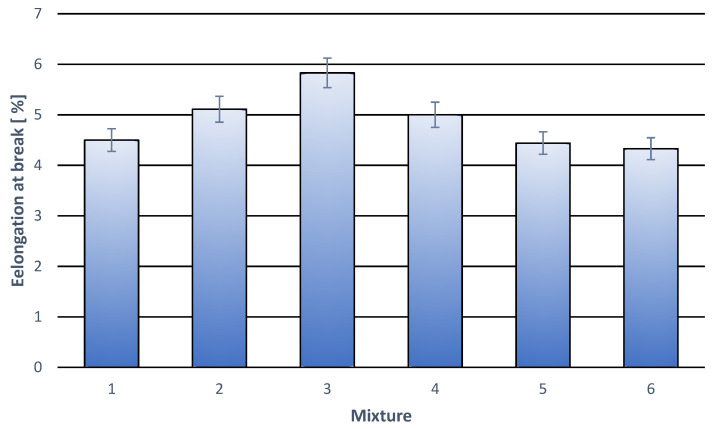
Elongation at break for individual polymer compositions. (**1**) 100% wt. PLA; (**2**) 97% wt. PLA + 3% wt. modifier; (**3**) 95% wt. PLA + 5% wt. modifier; (**4**) 92% wt. PLA + 3% modifier + 5% wt. cork filler; (**5**) 87% wt. PLA + 3% wt. modifier + 10% wt. cork filler; (**6**) 82% wt. PLA + 3% modifier + 15% cork filler.

**Figure 3:**
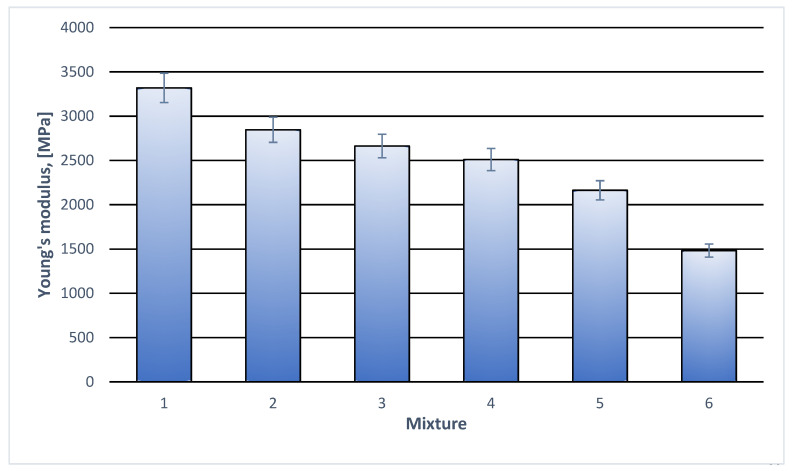
Young’s modulus of individual polymer compositions. (**1**) 100% wt. PLA; (**2**) 97% wt. PLA + 3% modifier; (**3**) 95% wt. PLA + 5% wt. modifier; (**4**) 92% wt. PLA + 3% modifier + 5% wt. cork filler; (**5**) 87%wt. PLA + 3% modifier + 10% wt. cork filler; (**6**) 82% wt. PLA + 3% modifier + 15% wt. cork filler.

Tensile stretching curves for all tested compositions had the same rectilinear character till the break of the specimen. No yield stress or any deviations from the rectilinear plot were observed. That is why the curves are considered a little interesting and thus they are not presented in this work. They bring, however, much key information concerning the mechanical properties of the material. Based on the curves, important parameters were determined. They were stress at break, relative deformation at break and Young’s modulus. The results are illustrated in Table 3 and in Figure 1, Figure 2 and Figure 3.

Initial studies focused on assessing the effect of modifier content on the properties of polylactide (PLA). The results (items 1–3, Table 3) showed that increasing the modifier content above the recommended concentration of 3% wt. is unjustified. At a concentration of 5% wt., a slight deterioration in some mechanical parameters, tensile strength and Young’s modulus, was observed. Only elongation improved slightly, but the value is within the margin of error. This confirms that higher concentrations not only do not provide measurable benefits but can negatively impact the material’s properties. Therefore, further testing was limited to samples containing 3% wt. modifier with varying amounts of cork filler.

The strength of the material decreased with the increased cork content (Figure 1). It is interesting, however, that the decrease was not as rapid as it is in some other works, so this kind of composition is not disqualified from further investigation.

The results concerning elongation at break indicate an interesting dependence on the content and kind of applied additives. They can be seen in Table 3 and in Figure 2. In the case of compositions containing only a modifier, an important ability to deform is observed. For the 5% wt. modifier content the elongation is close to 6%, which points to good cooperation with the polymer base. Introducing cork filler causes lowering of this value; the drop is, however, not important. For 15% wt. filler content and 3% wt. modifier content, elongation at break remains at the level like the one for pure PLA. The obtained results indicate an advantageous effect of both filler and modifier on mechanical properties and show good interfacial compatibility in the PLA-based composites.

In the case of Young’s modulus, a systematic drop can be seen both with the increased filler content and increased modifier content. The value of Young’s modulus for pure PLA is about 3.400 MPa both given in the manufacturer’s catalogue and resulting from the measurements. The essential decrease in the values is observed only at 10% wt. cork filler. It can be assumed that both the modifier and small quantities of the cork improve deformability of the specimens (Table 3, Figure 2). The detailed results for Young’s modulus are presented in Table 3 and Figure 3.

**Figure 4:**
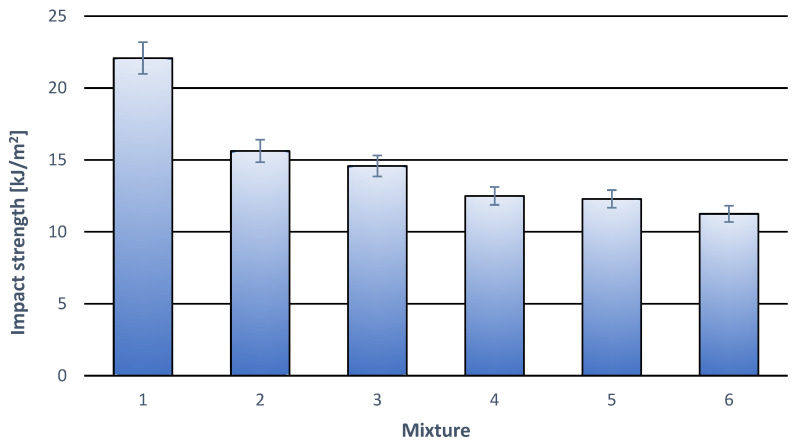
Impact strength of individual polymer compositions. (**1**) 100% wt. PLA; (**2**) 97% wt. PLA + 3% wt. modifier; (**3**) 95% wt. PLA + 5% wt. modifier; (**4**) 92% wt. PLA + 3% wt. modifier + 5% wt. cork filler; (**5**) 87% wt. PLA + 3% wt. modifier + 10% wt. cork filler; (**6**) 82% wt. PLA + 3% modifier + 15% wt. cork filler.

The results of the impact strength tests are presented in Table 4 and Figure 4. The measurements took place on double the number of specimens due to significant dispersion of the results. Generally, it can be stated that both the additive of modifier and the cork filler cause the decrease in this parameter compared to pure PLA. As is in the case of static tensile tests, for the specimens containing solely the modifier, the measured values of the impact strength are small and within the margin of error. Only introducing cork filler brings about noticeable drop of impact strength. What is worth noticing, the content of the filler within the range 5% wt. to 15% wt. does not influence a further drop in the impact strength, which remains at a level about 1 kJ/m^2^. The results suggest that the modifier improves interfacial adhesion between the cork filler and the PLA base, which limits the negative influence of the cork filler on the impact strength of the composite.

**Figure 5:**
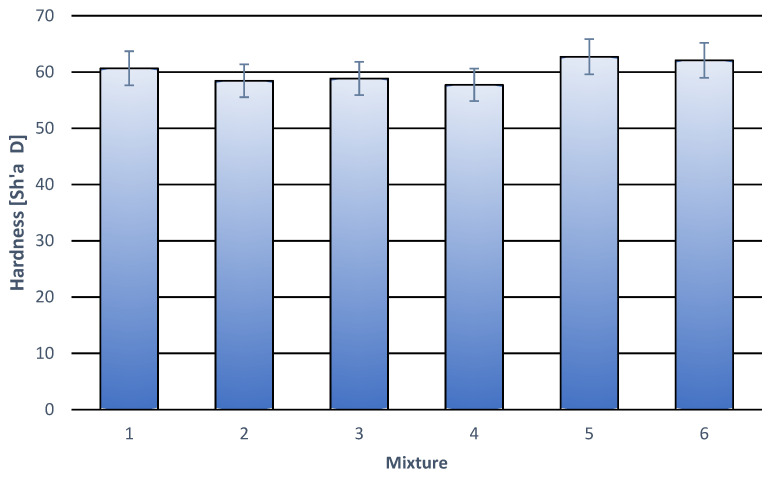
Hardness of individual polymer compositions. (**1**) 100% wt. PLA; (**2**) 97% wt. PLA + 3% wt. modifier; (**3**) 95% wt. PLA + 5% wt. modifier; (**4**) 92% wt. PLA + 3% wt. modifier + 5% wt. cork filler; (**5**) 87% wt. PLA + 3% wt. modifier + 10% wt. cork filler; (**6**) 82% wt. PLA + 3% modifier + 15% wt. cork filler.

The hardness of the tested compositions (Table 3, Figure 5) remains close to that of pure PLA, ranging from 57 to 62 degrees in the Shore D scale. All obtained results are within the measurement error range, demonstrating the stability of the mechanical properties of the analyzed materials. Among all the samples, those containing 10% wt. and 15% wt. of cork, and 3% wt. of modifier stand out, showing slightly higher hardness values. This phenomenon can be considered beneficial, although its mechanism has not been fully explained. The most likely explanation is a synergistic interaction between the system components, in which the modifier (compatibilizer) improves interfacial adhesion between the polymer matrix and the cork filler particles. As a result, although cork, as a material with lower hardness, should theoretically reduce this property, stabilization and even a local increase in the composition hardness is observed. These results confirm the good compatibility of the polymer matrix and cork filler system, as well as the effectiveness of the modifier in improving the material’s structural integrity. This may be important for further optimizing the functional properties of PLA-based composites.

**Figure 6:**
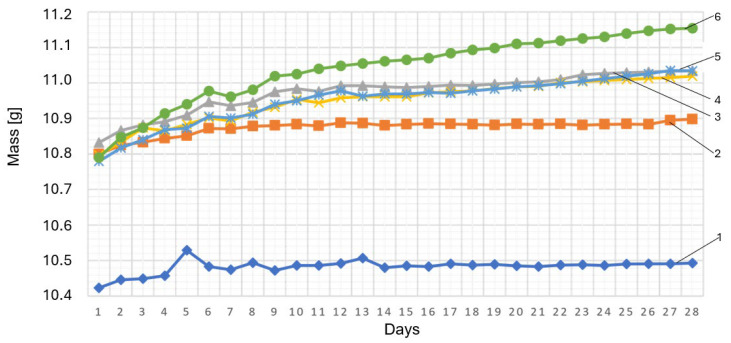
Weight change in samples subjected to the water soaking process (absorption measurement). (**1**) 100% wt. PLA; (**2**) 97% wt. PLA + 3% wt. modifier; (**3**) 95% wt. PLA + 5% wt. modifier; (**4**) 92% wt. PLA + 3% wt. modifier + 5% wt. cork filler; (**5**) 87% wt. PLA + 3% wt. modifier + 10% wt. cork filler; (**6**) 82% wt. PLA + 3% wt. modifier + 15% wt. cork filler.

From the biodegradation point of view, the key factor is the material’s ability to absorb water, which is one of the main initiators and accelerator of biodegradation both in the natural environment and in the industrial composting. This parameter was determined according to ISO 62. The range of the testing was widened by measuring (weighing) the mass of the specimens submerged in water for a period of 28 days. The results are presented in Figure 6.

The results show that the presence of cork in the polymer base causes a significant increase in the absorbing ability of the material, which is an expected phenomenon because of the natural porous structure and the hydrophilic character of the filler. The increased water absorption may be the result of the absorption ability of the cork in its micro pores and creation of micro fissures at phase boundaries, which promotes penetration of the liquid into the material volume.

It is astonishing to see the increase in absorption in the case of the specimens containing a sole modifier without the cork additive. This phenomenon may suggest that this additive, due to its chemical properties, influences the increase in electrostatic polarity of the polymer base or causes some changes in its morphology like the increase in free volume or the creation of surface irregularities. Finally, the creation of a bigger number of active centers is possible, which makes adsorption and diffusion of water easier.

The obtained results indicate that the presence of cork filler and modifier may in different ways modify hydrophilic properties of PLA-based composites, which is essential for their further degradation in humid environments. Table 5 illustrates the percentage of water absorption for the applied polymer compositions.

The next stage of the research involved structural analysis using Fourier Transform Infrared Spectroscopy, aimed at identifying and assessing potential chemical interactions between the PLA polymer matrix, the cork filler and the modifier. The measurement was conducted within a wavenumbers range of 4000 to 500 cm^−1^, which corresponds to the standard analytical range for organic polymer systems and enables the observation of characteristic vibrational bands of both the PLA functional groups and the additional components.

The measurement results were presented as absorption spectra, with band intensity values plotted against wavenumber. The positions and relative intensities of characteristic peaks were identified for each material composition, allowing for a comparison of changes in the chemical structure of the system resulting from the modification. Particular attention was paid to the band regions corresponding to the stretching vibrations of carbonyl groups (approx. 1750 cm^−1^), hydroxyl groups (approx. 3300–3500 cm^−1^) and the 1000–1200 cm^−1^ range, characteristic of C-O-C bond vibrations in the polyester structure. Analysis of these regions allowed for the assessment of whether new chemical bonds or physical interactions, such as hydrogen bonds, occurred between the composition components, indicating increased interfacial compatibility.

The measurement range of 4000–500 cm^−1^ is fully accurate and consistent with typical FTIR analysis conditions for polymeric materials. It allows for the recording of all important bands characteristic of functional groups present in the PLA and cork structures, as well as potential products of their interactions with the modifier [63,64,65,66].

**Figure 7:**
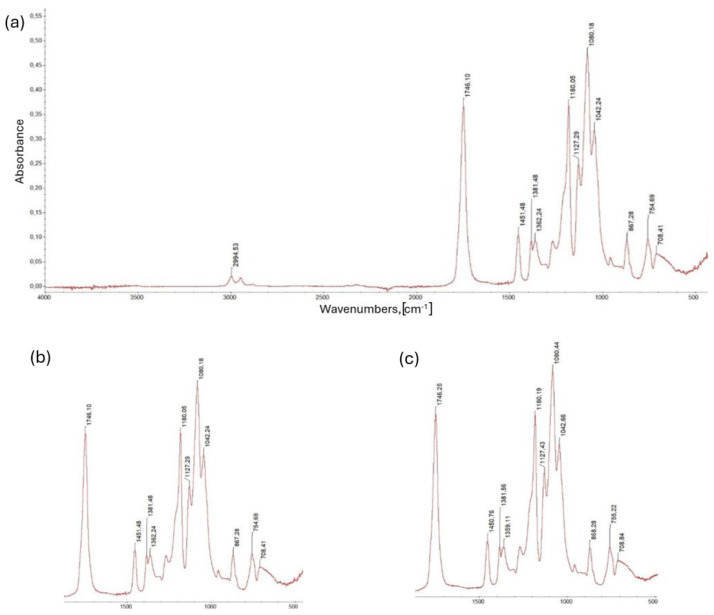
FTIR spectra of individual PLA samples with modifier. (**a**) Sample from PLA itself. (**b**) Spectrum of a sample with a composition of 97% wt. PLA and 3% wt. modifier. (**c**) Spectrum of a sample is composed of 95% wt. PLA and 5% wt. modifier.

Figure 7a presents the FTIR spectrum of pure polylactide (PLA), which clearly shows characteristic vibrational bands corresponding to its chemical structure. The strongest peak at 1746 cm^−1^ corresponds to stretching vibrations of the C=O bond in the ester group, which is the basic structural element of polylactide. In the range of 1250–1050 cm^−1^, intense bands associated with vibrations of C-O-C and C-O bonds are observed, characteristic of ester bonds present in the PLA polymer chain. The presence of numerous, well-resolved peaks in this range indicates a high degree of order in the polymer structure, which may indicate the partially crystalline nature of the material under study. However, in the 3200–3600 cm^−1^ region, there are no distinct absorption bands originating from stretching vibrations of hydroxyl (-OH) groups, confirming that the pure PLA sample does not contain hydrophilic impurities such as water, cellulose, or starch. Spectral analysis therefore confirms the typical chemical structure of PLA and provides a reference point for further comparisons with compositions containing a cork filler and a modifier.

Figure 7b shows the FTIR spectrum of a sample containing 97 wt.% PLA and 3 wt.% modifier. Spectral analysis shows the retention of all characteristic bands typical of pure PLA, including the dominant band corresponding to the stretching of the C=O carbonyl bond at 1746 cm^−1^.

In the 1000–1200 cm^−1^ range, some changes in the intensity and shape of the bands are observed, suggesting the presence of additional C-O bonds, likely resulting from the introduction of the modifier into the composition. However, the 3200–3600 cm^−1^ region remains free of signals characteristic of hydroxyl groups (-OH), indicating the absence of water or other hydrophilic impurities and indicating good compatibility and integration of the modifier with the polymer matrix.

The spectrum therefore confirms that the modifier does not cause degradation or significant structural changes in PLA, and its presence can only influence local chemical interactions within the ester bonds.

The next measurement was performed on a sample containing 95% wt. PLA and 5% wt. modifier. The FTIR spectrum in this case shows noticeable differences compared to pure PLA (Figure 7c). The band corresponding to the stretching of the carbonyl C=O bond at 1746 cm^−1^ is still dominant, but the range 1000–1200 cm^−1^ has become broader and more intense, indicating an increased share of C-O bonds associated with the presence of the modifier. Additionally, the emerging band around 3300 cm^−1^ may suggest the presence of hydroxyl groups (-OH), which are absent in the spectrum of pure PLA. The observed changes confirm the correct incorporation of the modifier into the composition and its effect on the local chemical structure of the material, including the formation of additional polar bonds and possible interfacial interactions in the polymer–modifier system.

**Figure 8:**
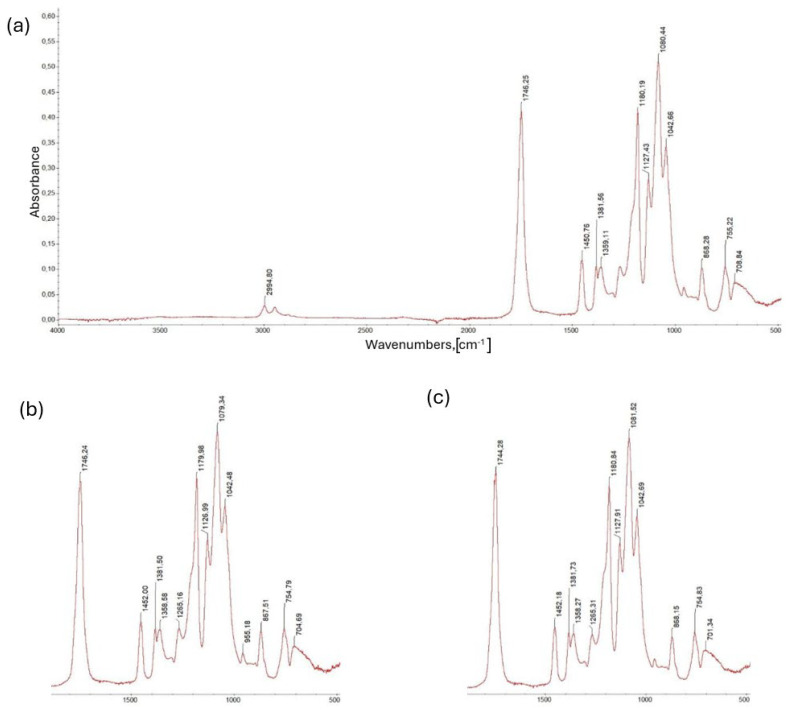
Spectra of individual PLA polymer compositions with cork filler: (**a**) 92 wt.% PLA, 5 wt.% cork filler and 3 wt.% modifier; (**b**) 87 wt.% PLA, 10 wt.% cork filler and 3 wt.% modifier; (**c**) 82 wt.% PLA, 15 wt.% cork filler and 3 wt.% modifier.

Figure 8a shows the FTIR spectrum of a sample containing 92% wt. PLA, 5% wt. cork filler and 3% wt. modifier. It confirms the presence of all components of the composition. The characteristic stretching band of the carbonyl C=O bond at 1746 cm^−1^ remains preserved, while the range of 1000–1200 cm^−1^ has clearly broadened, indicating overlapping vibrations of the PLA ester bonds with additional C-O bonds originating from the modifier and cork. The presence of hydroxyl groups is visible as a diffuse band in the region of approximately 3300 cm^−1^, which is characteristic of naturally derived materials such as cork. The spectrum suggests that the components of the composition were physically combined, without chemical reactions or degradation of the PLA structure. The obtained results confirm good interfacial compatibility in the polymer–filler–modifier system.

Figure 8b shows the FTIR spectrum of a composition containing 87 wt.% PLA, 10 wt.% cork filler and 3 wt.% modifier. The characteristic ester band at 1746 cm^−1^, corresponding to the stretching vibrations of the C=O bond in the PLA structure, is preserved. In the range of 1000–1200 cm^−1^, a clear broadening and increased intensity of the bands is observed, indicating the presence of additional C-O bonds resulting from the participation of a cork and modifier in the composite structure.

A diffuse hydroxyl band appears around 3300 cm^−1^, confirming the presence of -OH groups characteristic of naturally occurring components. Spectral analysis demonstrates good integration of the organic phase (cork) with the polymer matrix, with no signs of chemical degradation of the PLA. The obtained results indicate good compatibility and structural stability of the polymer–filler–modifier system.

The last material composition analyzed, shown in Figure 8c, is a sample containing 82% wt. PLA, 15% wt. cork filler and 3% wt. modifier. The spectrum for this sample is shown in Figure 8. In this composition, it shows the presence of a characteristic C=O ester band at 1746 cm^−1^, typical of the polylactide structure. In the range of 1000–1200 cm^−1^, a broad and intense band is visible, resulting from the overlap of C-O-C bond vibrations in the PLA chain and signals originating from the cellulose–lignin fraction of the cork.

Additionally, a broad hydroxyl band is observed in the region around 3300 cm^−1^, confirming the presence of -OH groups characteristic of plant-derived components. The obtained spectrum clearly indicates a high proportion of the natural fraction in the composite, while maintaining the chemical integrity and structural stability of the PLA matrix. These results confirm that the modifier used promotes effective bonding of the polymer phase with the cork filler without degrading the base material.

## 4. Conclusions

Based on the obtained results it can be stated that the produced PLA-based compositions containing powdered cork and a modifier (compatibilizer) present an innovative, fully degradable material of advantageous utility and environmental properties. The results confirm the assumption of comprehensive validity of PLA modification with natural filler.

Introducing powdered cork to the PLA base, despite the decreased values of both strength and Young’s modulus, does not disqualify the material. It is particularly useful in the packaging industry where the strength requirements are not very strict. The decrease in the impact strength is also kept within acceptable limits.

An interesting and positive phenomenon is the increase in hardness at higher cork content, which points to synergic interaction between the filler and the polymer base.

The increased water absorption, being the result of porous structure of the cork, could possibly accelerate biodegradation, but the observations show that the water absorption takes place mainly in the cork structure while the PLA base remains intact. This kind of behavior makes a difference between cork and typical lignocellulosic fillers (e.g., cellulose fibers) giving it unique properties.

From the environmental and industrial point of view this composition can be considered as highly innovative material. Linking a biodegradable base with natural waste (cork) enables us to obtain a product of low manufacturing cost with simultaneous use of renewable, difficult to utilize waste material.

Summing up, the composition worked out and presented in this paper: PLA–cork-modifier joins biodegradability, ecological character, chemical stability and satisfying mechanical properties, which makes it a prospective and innovative material for packaging industry and many other pro-ecological applications.

## Figures and Tables

**Table 1:** Composition of individual polymer compositions.

No.	PLA [% wt.]	Cork Filler [% wt.]	Modifier [% wt.]
1	100	-	-
2	97	-	3
3	95	-	5
4	92	5	3
5	87	10	3
6	82	15	3

**Table 2:** Injection process parameters.

Injection Parameters		Values
Injection:		
Speed		40 mm/min
Pressure		190 bar
Processing Temperature	zone 1	185 °C
	zone 2	185 °C
	zone 3	180 °C
	zone 4	180 °C
	zone 5	80 °C
Pressurize		
Time		10 s
Clamping Pressure		40 bar
Closing Force		
Average		844 N
Closing the Mold		
Pressure		170 bar
Speed		35%
Mold Protection Time		10 s
Cycle Time		122 s
Counter Pressure		5 bar
Opening the Mold		
Counter Pressure		10 bar
Cooling Time		30 s
Temperature		40 °C

**Table 3:** Results of the tensile stretching tests (M–modifier, K–cork filler).

No.	Material	Maximum Tension [MPa]	Elongation at Break [%]	Young’s Modulus [MPa]
1	100% wt. PLA	67.90 ± 1.42	4.50 ± 1.3	3319.56 ± 31.66
2	97% wt. PLA + 3% wt. M	66.13 ± 1.00	5.11 ± 0.12	2846.60 ± 19.52
3	95% wt. PLA + 5% wt. M	58.50 ± 4.06	5.83 ± 0.13	2662.79 ± 84.06
4	92% wt. PLA + 5% wt. K + 3% wt. M	38.87 ± 3.59	5.00 ± 0.20	1710.13 ± 71.87
5	87% wt. PLA + 10% wt. K + 3% wt. M	33.87± 2.29	4.44 ± 0.12	2163.11 ± 66.42
6	82% wt. PLA + 15% wt. K + 3% w. M	29.20 ± 0.99	4.33 ± 0.20	1482.47 ± 22.92

**Table 4:** Impact strength and hardness of individual polymer compositions.

No.	Material	Impact Strength [kJ/m^2^]	Hardness, Sh’a “D”
1	100% wt. PLA	22.08 ± 1.38	60.66 ± 1.93
2	97% wt. PLA + 3% wt. M	15.62 ± 1.19	58.45 ± 1.09
3	95% wt. PLA + 5% wt. M	14.58 ± 0.58	58.85 ± 0.93
4	92% wt. PLA + 5% wt. K + 3% wt. M	12.50 ± 0.85	57.71 ± 1.32
5	87% wt. PLA + 10% wt. K + 3% wt. M	12.29 ± 0.72	62.71 ± 1.23
6	82% wt. PLA + 15% wt. K + 3% w. M	11.25 ± 0.73	62.07 ± 0.89

**Table 5:** Change in water absorption for individual material compositions.

Water Absorption [%]						
No.	Material	1 Day	7 Days	14 Days	28 Days	
1	100% wt. PLA	0	0.49	0.55	0.67	±0.02
2	97% wt. PLA + 3% wt. M	0	0.66	0.74	0.91	
3	95% wt. PLA + 5% wt. M	0	0.96	1.45	1.86	
4	92% wt. PLA + 5% wt. K + 3% wt. M	0	0.89	1.52	2.06	
5	87% wt. PLA + 10% wt. K + 3% wt. M	0	1.13	1.76	2.37	
6	82% wt. PLA + 15% wt. K + 3% wt. M	0	1.58	2.51	3.37	

## Data Availability

The original contributions presented in this study are included in the article. Further inquiries can be directed to the corresponding author.
